# Addressing Sexually Transmitted Disease Management Through Practitioner Education on Florida Reporting Requirements in Manatee County: A Quality Improvement Initiative

**DOI:** 10.7759/cureus.113853

**Published:** 2026-08-03

**Authors:** Christina N Bobrek, Claudia R Silver, Zade Moureiden, Cynthia Samra, Anastasia Peele

**Affiliations:** 1 College of Medicine, Florida State University College of Medicine, Sarasota, USA; 2 Department of Health in Manatee County, Florida Department of Health, Bradenton, USA

**Keywords:** florida counties, healthcare practitioner, public health education, sexually transmitted disease (std), underreporting

## Abstract

Introduction: Successful public health interventions depend on accurate disease surveillance, which can be hindered by inconsistent reporting of confirmed cases of disease by practitioners. This quality improvement project aimed to develop an intervention that will lead to an increase in the number of practitioner disease reporting forms (PDRFs) submissions to the Florida Department of Health in Manatee County (DOH-Manatee) for all sexually transmitted disease and infection (STD/STI)-positive laboratory results.

Methods: Following the Standards for Quality Improvement Reporting Excellence (SQUIRE) 2.0 Guidelines, the Plan-Do-Study-Act methodology was utilized and led to the use of a mixed-methods approach. The quantitative analysis included comparing the number of positive STD/STI tests to the number of submitted PDRFs. The qualitative data collection included surveys with Disease Intervention Specialists (DIS) at the DOH-Manatee to identify successful practices, challenges with practitioners, and factors preventing follow-up. Interventions included creation of an email and letter containing Florida Statutes reporting guidelines, standardized PDRFs, and clear submission instructions. The intervention is on hold, pending approval of the design and outreach plan by DOH-Manatee and the Florida Department of Health.

Results: Of the three DOH-Manatee DIS, two responded. They reported barriers to form submission, including limited practitioner education on reporting guidelines, insufficient patient contact information on laboratory reports, and lack of practitioners’ knowledge of DIS jurisdiction. Notably, both survey responders indicated uncertainty about tracking processes for positive laboratory results and PDRFs. Interventions that have been successful in the past included DIS members visiting practitioner offices and speaking with them directly. Per DOH-Manatee DIS, Manatee County had 196 positive tests, but only 45 PDRFs (23%) were faxed in July 2025.

Discussion: Underreporting of PDRFs impacts efforts to control rates of STDs/STIs by delaying patient treatment and follow-up services. Limitations included restricted access to DOH-Manatee data, switching from focus group to survey methodology, and leadership turnover affecting internal operations.

Conclusion: Stronger practitioner education and collaboration between healthcare systems and public health should enhance reporting compliance and reduce ongoing transmission. Planned interventions include targeted practitioner education on Florida Statutes reporting requirements and clarification of practitioner roles in disease transmission prevention. These outreach efforts will be continued internally within the quality improvement (QI) division of the DOH-Manatee.

## Introduction

Sexually transmitted diseases and infections (STDs/STIs) pose a significant public health challenge in Florida. In 2024, the rate of STDs/STIs per 100,000 people in Florida was 724.5, with Manatee County (MC) exhibiting higher rates of STDs/STIs compared to three peer counties in 2024 [1]. The rates per 100,000 people were 520.2, 366.3, 483.2, and 436.2 for Manatee, Pasco, Seminole, and Collier counties, respectively [1]. Ensuring timely reporting of STD/STI cases to the Florida Department of Health (FLDOH) is necessary for notification of partners, verification of treatment, and disease surveillance.

In the United States, there continues to be an increase in STIs each year, with over 2.5 million cases of gonorrhea, chlamydia, and syphilis reported by the Centers for Disease Control and Prevention (CDC) in 2022 [2]. In women, STIs can lead to more serious complications, including pelvic inflammatory disease, infertility, and ectopic pregnancy [2]. This is especially concerning because STIs can often be asymptomatic, and over 1 million new STIs are contracted daily worldwide [3]. Diagnosis, reporting, treatment, and follow-up for STDs/STIs are essential to prevention of further transmission and complications.

There is a discrepancy between the number of STD/STI-positive laboratory reports and practitioner disease reporting forms (PDRFs) submitted. When patients test positive for an STD/STI, such as chlamydia, the laboratory report result is automatically sent to the Florida Department of Health in Manatee County (DOH-Manatee). However, the PDRF is a separate document that must be filled out by the practitioner to provide contact information [4]. PDRFs allow the DOH-Manatee to reach out to patients and inform them of best practices for preventing further disease, if needed. Per DOH-Manatee Disease Intervention Specialists (DIS), MC had 196 positive tests, but only 45 PDRFs were faxed in July 2025, suggesting gaps in reporting and follow-ups. Each positive test is expected to generate a unique PDRF submission. Those responsible for submitting PDRFs include any licensed allopathic, osteopathic, chiropractic, naturopathic, or veterinary practitioner who is actively practicing [5].

This quality improvement project aimed to develop an intervention that will lead to an increase in the number of PDRF submissions to the DOH-Manatee for all STD/STI-positive laboratory results. This goal was to be accomplished by identifying the DIS department’s perceptions of the barriers within the DOH-Manatee as well as their perceptions of providers’ barriers to completing PDRFs. Then, an educational program would be developed to address these issues.

## Materials and methods

Context 

MC has a high STD/STI burden when compared to peer counties, emphasizing the need for intervention. There are known discrepancies between positive laboratory results and submitted PDRFs. This underreporting is concerning, as practitioners have a legal obligation to report any diseases included in the reportable diseases outlined by the FLDOH in a timely fashion. The lack of a standardized tracking system further complicates the issue, as there is poor workflow between practitioners and DOH-Manatee. The DOH-Manatee felt there may have been knowledge gaps concerning practitioner reporting requirements and the role of the DIS within the DOH-Manatee. Many practitioners still rely on faxing and manual systems for reporting, further limiting compliance. The DOH-Manatee had noticed that Federally Qualified Health Centers (FQHCs) and community clinics are among the highest reporters, while others such as emergency rooms, urgent cares, and private practices rank among the lowest.

Interventions

The intervention was designed to educate practitioners on the state of Florida reporting requirements for STD/STIs. Materials included a standardized practitioner letter outlining Florida STD/STI laws regarding reportable diseases and timelines. Additionally, an informational packet included clear instructions that outlined how to submit PDRFs by both electronic and fax submissions. A succinct PowerPoint presentation geared toward practitioners summarizing the reporting requirements and the public health impact of incomplete reporting was the last component of the packet. The role of DIS was described in detail. This intervention will be distributed through targeted outreach by the DOH-Manatee to practitioners, especially those with high-volume locations such as community clinics, obstetrics and gynecology (OB/GYN) offices, and urgent care centers in MC.

The team involved in this quality improvement project consisted of three third-year medical student investigators at the Florida State University College of Medicine. They were responsible for project design, data analysis, intervention development, and materials. This project was under the guidance and supervision of the DOH-Manatee Community Health Director. Other public health partners from within the DOH-Manatee included DIS and epidemiology staff who provided current STD/STI tracking data, insight into workflow, and survey data on reporting gaps and proposed interventions.

Study of the interventions

**Figure 1:**
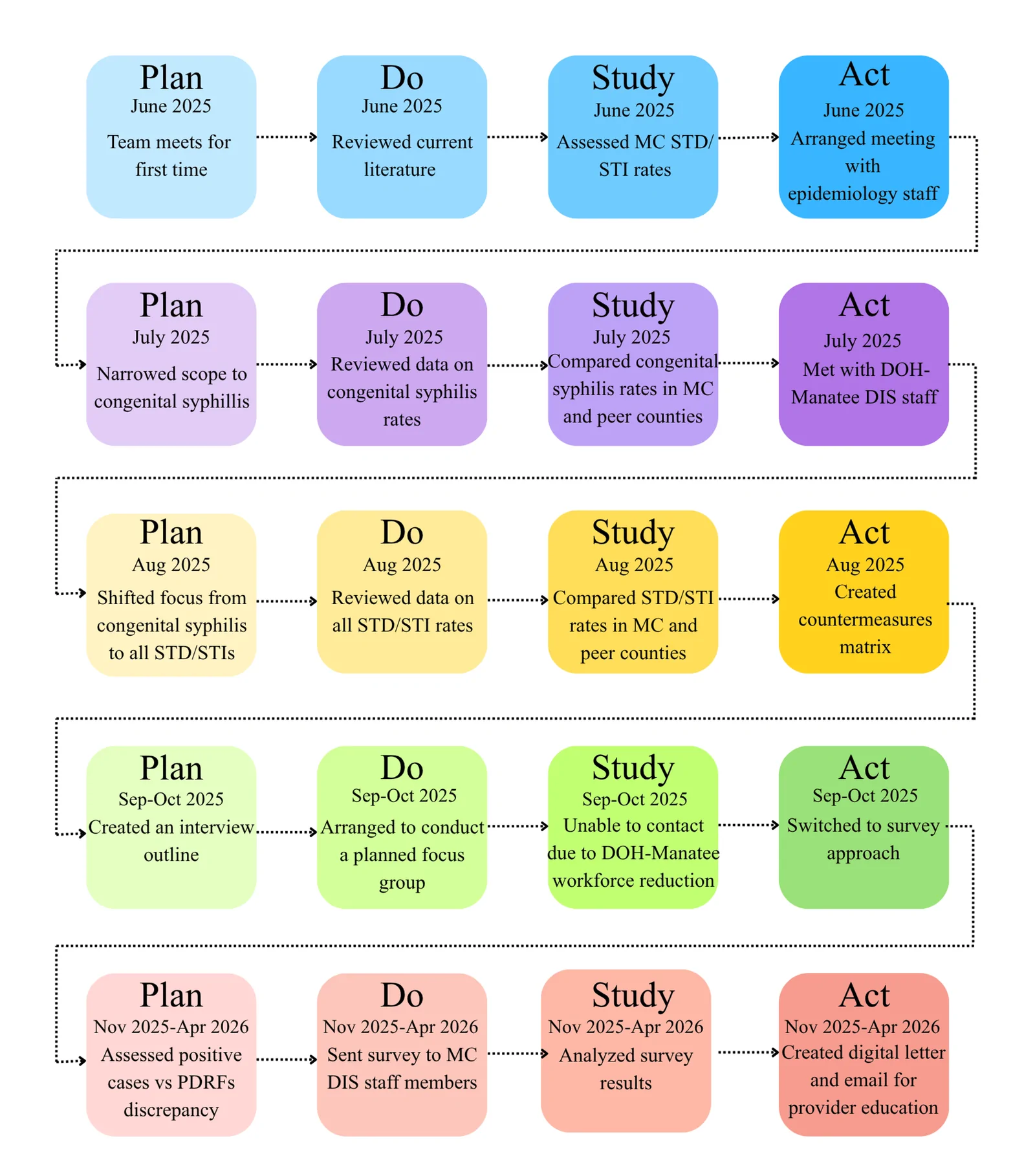
Plan-Do-Study-Act summary MC = Manatee County; STDs/STIs = sexually transmitted diseases and infections; DOH-Manatee = Florida Department of Health in Manatee County; DIS = Disease Intervention Specialists; PDRFs = practitioner disease report forms

A mixed-methods approach was utilized for this study, guided by a Plan-Do-Study-Act framework following the Standards for Quality Improvement Reporting Excellence (SQUIRE) 2.0 Guidelines (Figure 1) [6]. Quantitative analysis included comparing the number of positive tests to the number of submitted PDRFs. A qualitative assessment was performed and involved sending a survey to DIS to identify specific areas of concern, such as lack of patient contact information, practitioner knowledge gaps, and lack of knowledge of a tracking process. 

The development of the intervention has been completed, but distribution is on hold, pending DOH-Manatee and FLDOH approval. Once the intervention has been fully implemented, the number of completed laboratory results compared to PDRFs will be re-evaluated to assess if distribution of practitioner materials decreased the discrepancy. This can be further stratified by practitioner type. Additionally, DIS surveys can be redistributed following dissemination of practitioner materials to assess their perspective on changes that have occurred as a result of the interventions. The project will be continued internally with DOH-Manatee.

Measures

The primary outcome measure utilized was the discrepancy between the proportion of positive laboratory STD/STI results reported and the number of PDRFs submitted to DOH-Manatee. This outcome measure was chosen because these results directly show compliance with Florida Statutes reporting requirements and allow for identification of gaps in practitioner reporting [5]. The operational definition is the number of submitted PDRFs for reportable STDs/STIs divided by the number of positive laboratory STD/STI cases, with a target goal of achieving greater than 50%.

**Figure 2:**
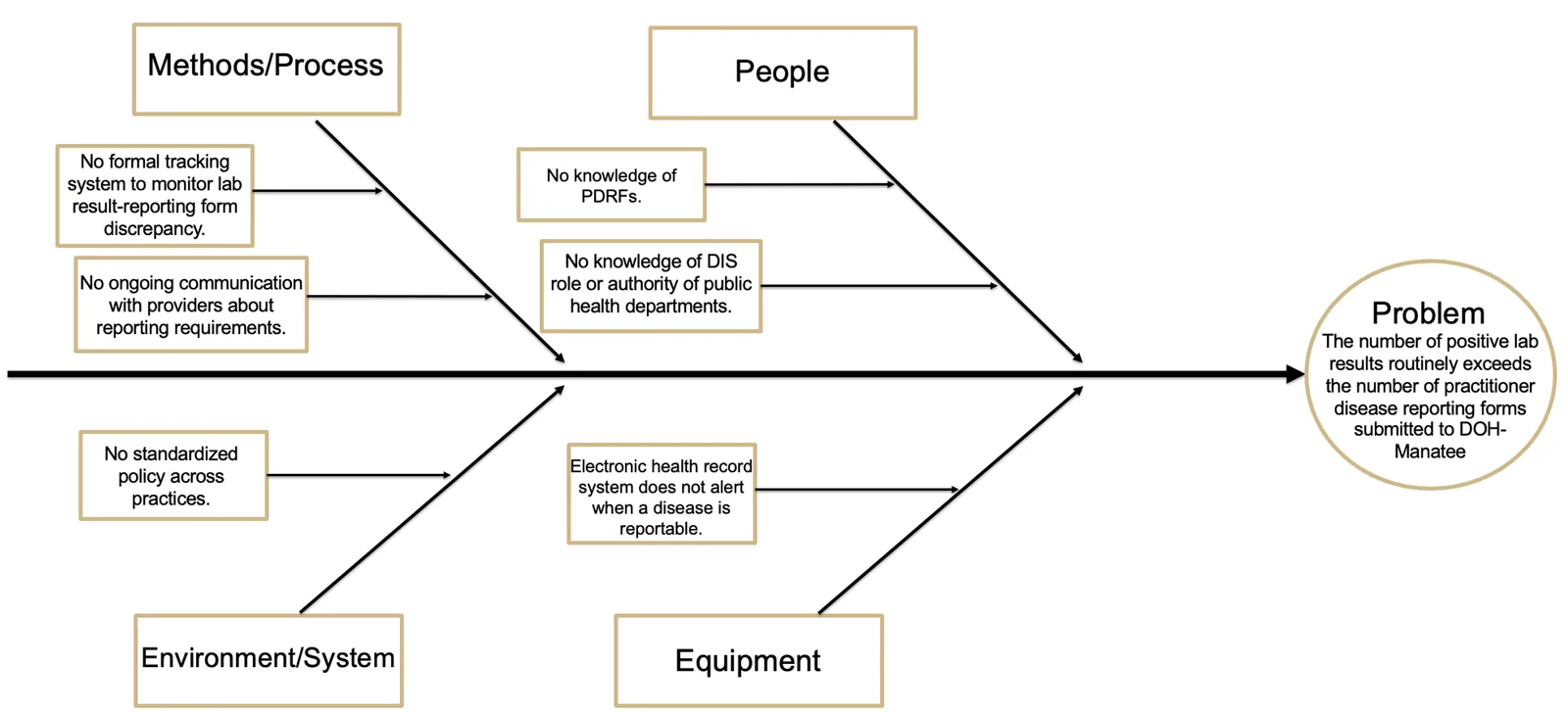
Fishbone diagram PDRFs = practitioner disease report forms; DOH-Manatee = Florida Department of Health in Manatee County; DIS = Disease Intervention Specialists

Our project approach of working with the DIS team gave us direct insight into the challenges they face in acquiring PDRFs (Figure 2).

**Figure 3:**
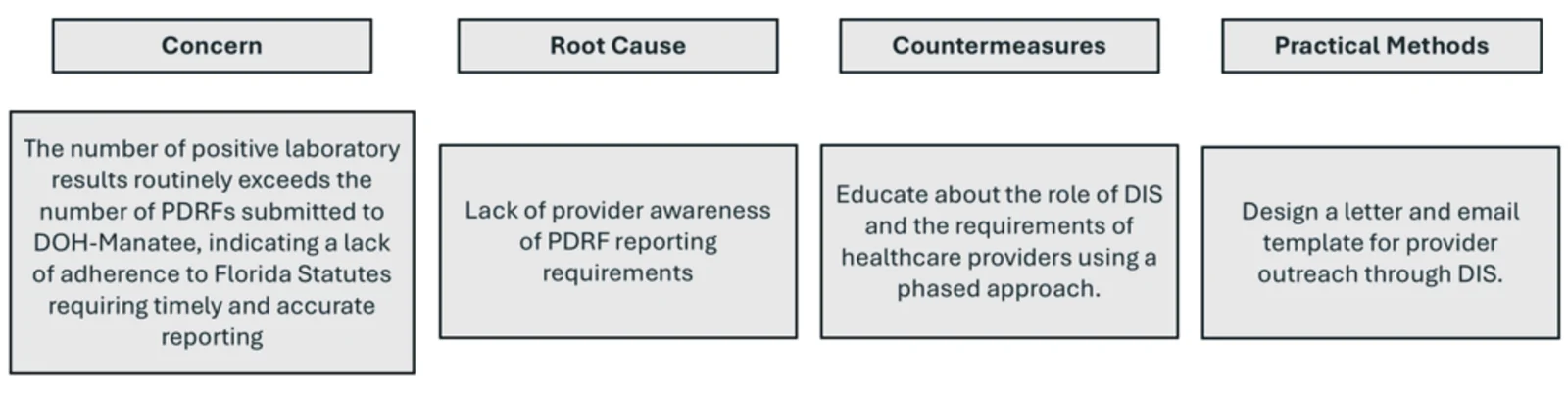
Countermeasure matrix explaining the methods behind the intervention PDRFs = practitioner disease report forms; DOH-Manatee = Florida Department of Health in Manatee County; DIS = Disease Intervention Specialists

This provided us with the information needed to decide on the digital letter and email outreach approach to increase practitioner awareness (Figure 3). Using the digital approach overcomes budget limitations and any barriers caused by DOH-Manatee employee shortages. Another approach considered was modifying the DOH’s internal tracking system; however, this was not feasible as it fell beyond the scope of the project.

Analysis

Qualitative analysis using Survey Monkey was performed (see Appendices). Results were used to direct the interventional approach. Future analysis will involve re-evaluation of the number of PDRFs that were submitted compared to positive laboratory results to assess if distribution of practitioner materials decreased the discrepancy.

Ethical considerations

Institutional Review Board approval was not required due to the study being in the pre-intervention stage, and this project did not include personally identifiable information. No conflicting relationships exist for any author.

## Results

Initial project efforts focused on identifying discrepancies between positive STD/STI laboratory results and PDRF submission in MC through review of surveillance data. A root cause analysis was conducted, which identified key contributors, most importantly practitioner knowledge deficits and communication barriers. The intervention was formed to target educational outreach to practitioners.

**Figure 4:**
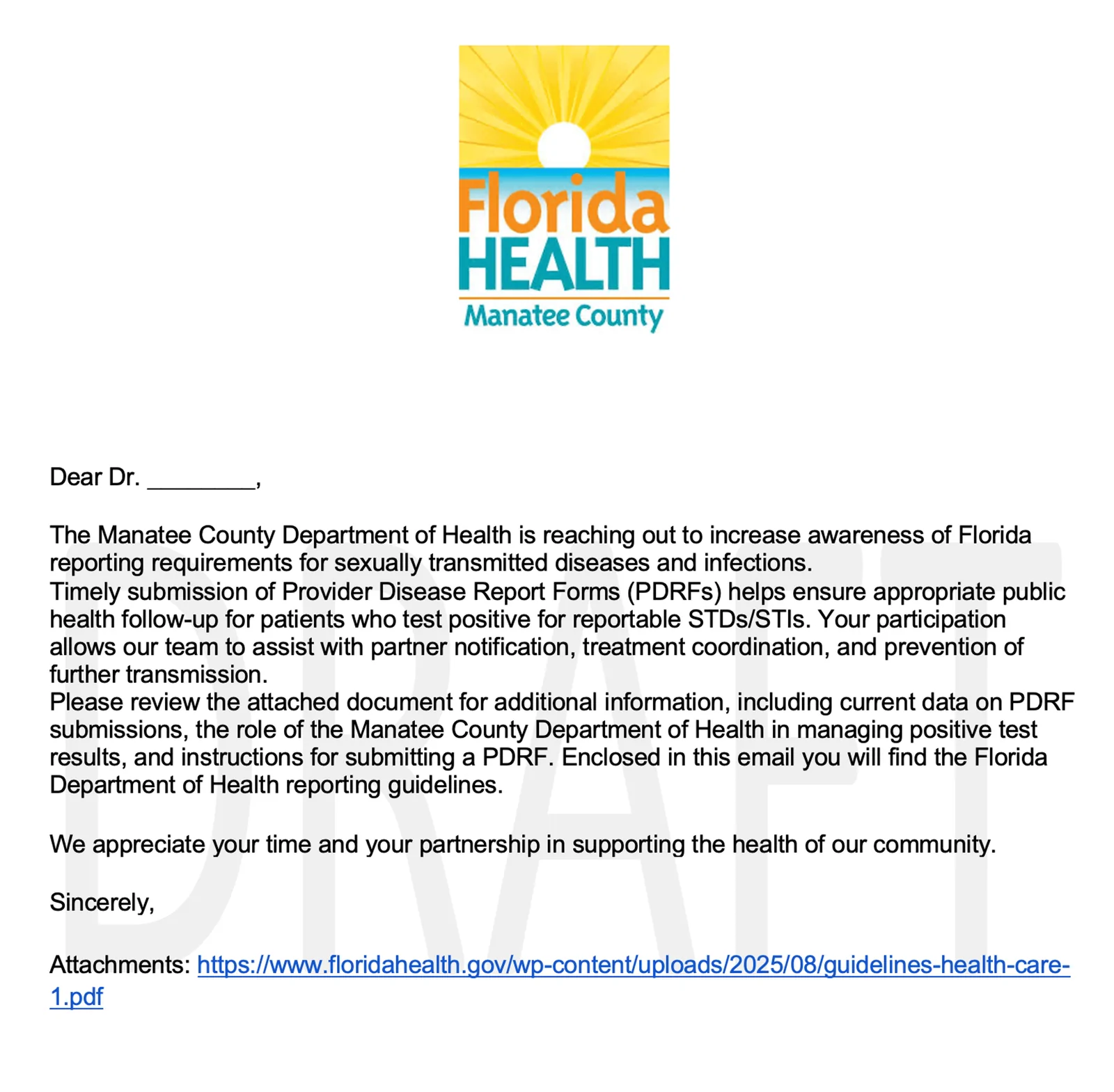
Draft letter template sent out to practitioners STDs/STIs = sexually transmitted diseases and infections

**Figure 5:**
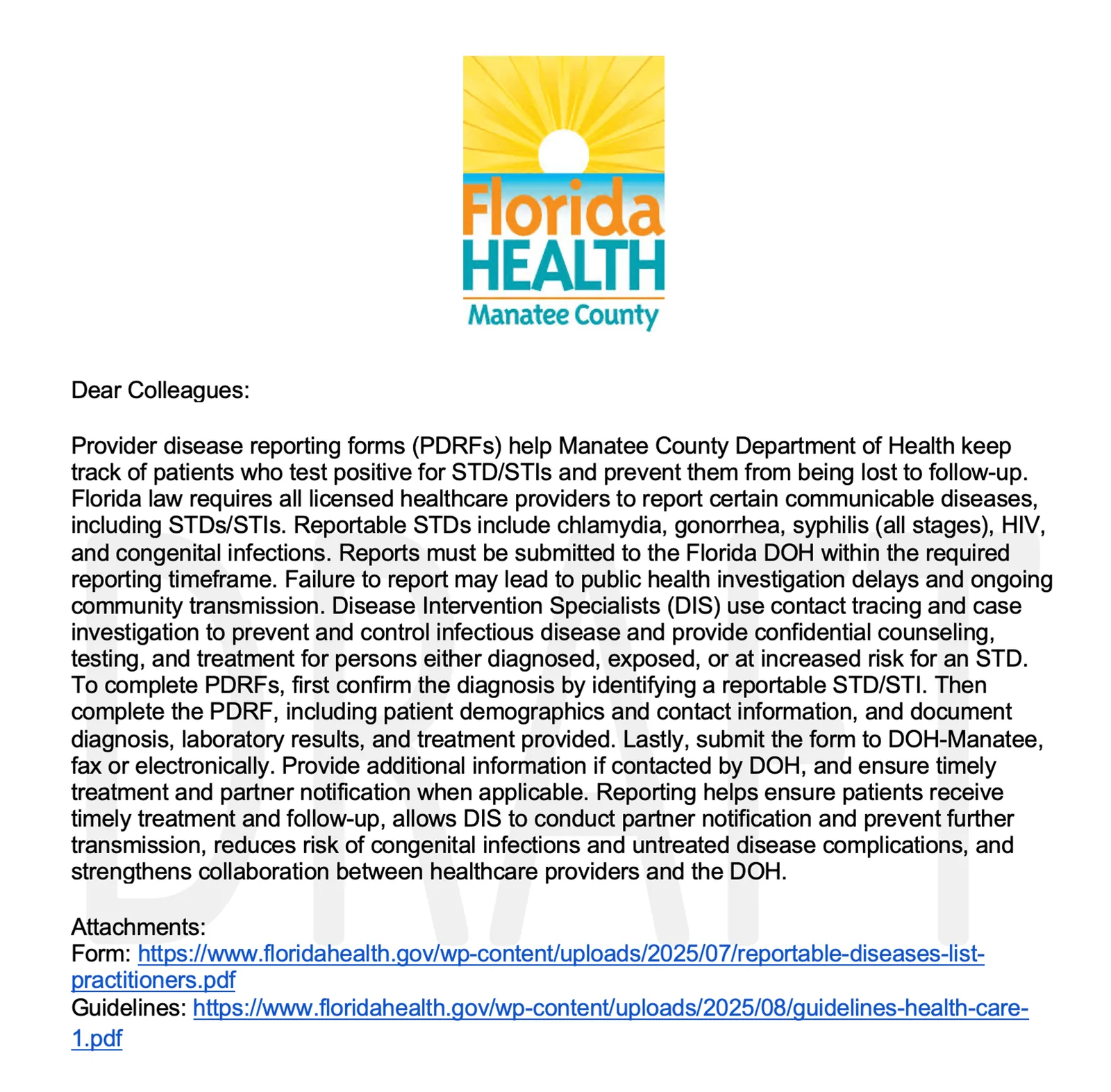
Draft email template sent out to practitioners STDs/STIs = sexually transmitted diseases and infections; HIV = Human Immunodeficiency Virus; DOH = Department of Health

The next steps were designing a practitioner-directed email or letter containing Florida Statutes reporting guidelines, standardized PDRFs, and clear submission instructions (Figures 4-5). The main distribution strategy will be focused on direct email outreach by DIS. No intervention has been distributed at this time as design and outreach plan approval are pending by DOH-Manatee and FLDOH.

DIS survey response results

Results from the qualitative survey were sent to the MC DIS to identify the barriers to submitting PDRFs. Two of the three DIS staff members responded. Conclusions regarding root causes are based on responses from only two individuals and therefore may not fully represent the perspectives of all relevant stakeholders. On a 1-5 scale (1 = terrible, 5 = excellent), DIS responders rated their relationships with practitioners as 2 and 5, respectively. Actions that have contributed to successful practitioner relationships included visiting the practitioner's office and establishing a point of contact. Interestingly, the DIS reported good communication and relationships with local OB/GYNs, but there is still a lack of PDRFs. 

Challenges with practitioner relationships included practitioners not knowing reporting responsibilities, not knowing the role of the FLDOH for reporting, and lack of trust in the FLDOH when handling private medical information. Successful practices that worked previously included conducting field visits to practitioner offices and speaking with them directly about the reporting requirements and FLDOH’s responsibilities. 

Both responders agreed they experience challenges with practitioners submitting the PDRFs. These included practitioners who were not aware that the PDRFs had to be completed or forms being filled incorrectly. Additionally, they noted that large facilities like hospitals do not submit PDRFs. When asked if the DIS tracks the discrepancy between PDRFs and positive laboratory results as a ratio, the DIS members responded “no” and “not sure.” Both members noticed there being a larger number of positive laboratory results than PDRFs submitted. One member acknowledged that part of the gap may be due to DIS receiving laboratory results faster than the practitioners. However, the other DIS member did not report this as a possible cause. Most of the PDRFs were completed at free community clinics and the local behavioral health hospital.

Current tracking of positive laboratory clients is done through the Syndromic Tracking and Reporting System (STARS) of the FLDOH. Initially, DIS only receives notification of a positive laboratory result. This necessitates DIS reaching out to practitioner offices to obtain any and all lacking PDRF information. On average each month, one member stated 85% of clients with positive laboratory results lack confirmed documentation of treatment, while the other reported 0%. When asked if they have difficulty getting the information they need from practitioners when following up on cases, the DIS members responded “yes” and “sometimes.” The difficulties they described included practitioners not knowing FLDOH guidelines and missing patient contact information. DIS noted that the PDRF includes information regarding patient treatment plans and other pertinent information.

When asked how often they need to send letters referencing Florida Statutes to request patient medical records from practitioners who are hesitant to share required information, the DIS members responded “often” and “sometimes.” Reasons for hesitancy to provide patient medical records included not knowing the FLDOH guidelines or regulations, lack of trust in the validity of DIS, and the mistaken belief that the Health Insurance Portability and Accountability Act of 1996 (HIPAA) [7] would be violated without patient consent.

## Discussion

Summary 

In summary, our survey findings identified provider education as a potential strategy to improve alignment between positive STD/STI laboratory results and PDRF submissions.

DIS staff described mixed relationships with healthcare practitioners, with one rating them as “poor” and another as “good.” Several challenges in practitioner relationships were noted, including lack of practitioner and staff knowledge regarding reporting responsibilities, difficulty contacting clinics, and limited trust in DIS when requesting patient information. Stronger relationships were often achieved by establishing a consistent point of contact within the clinic and through in-person outreach and communication of DOH roles and reporting requirements. OB/GYN practitioners were noted to have more consistent communication and testing practices. It may be helpful to further investigate their established workflows, which may be replicable in the future by other practices.

Successful strategies, such as establishing a designated point of contact and conducting in-person outreach, offer insight into best practices moving forward. While the intervention has been made but not distributed, this project successfully met its primary goal of identifying system and practitioner-level barriers to reporting compliance.

Based on survey responses, DIS perceived provider knowledge deficits as an important contributor to the lack of PDRF submissions. DIS respondents also identified potential communication barriers related to the role of DIS staff as public health partners who investigate and help prevent the spread of communicable diseases. Additionally, respondents reported that some practitioners may have concerns regarding HIPAA violations or patient consent requirements when sharing required records, which may contribute to hesitancy in submitting PDRFs. DIS respondents identified three primary issues related to PDRF submission: incomplete forms, incorrectly completed forms, and forms not being submitted. These findings suggest that, from the perspective of public health staff, limited awareness of reporting requirements and processes may contribute to discrepancies between positive laboratory results and submitted PDRFs. However, direct assessment of practitioner knowledge and practices is needed to confirm these factors as determinants of underreporting.

Strengths of the project included identifying the large discrepancy between the number of positive STD/STI tests versus the number of submitted PDRFs, and getting the perspective of the “why” from the DIS staff themselves. From this, we were able to create an educational intervention for practitioners that is low-cost, practical, and sustainable. We have left this quality improvement project at a stage where it will be continued internally within the QI division of the DOH-Manatee.

Interpretation

Due to the inability to implement the intervention, we cannot address outcomes or associations at this time. Additionally, there is no system in place that directly tracks the ratio of STD/STI-positive laboratory results to PDRF submission. Future tracking processes would be needed to evaluate the effectiveness of our intervention.

Comparison of the results with previous literature revealed a historical theme of discrepancy between positive laboratory results and required PDRFs submitted. Many root causes have existed for such discrepancies, including lack of awareness of the legal requirement to report, which diseases are reportable, and how or to whom to report [8]. According to prior literature, the issue of underreporting of required communicable diseases, alongside similar root causes, has been present since before 2002 [8]. This signifies that the healthcare system still has yet to completely tackle this obstacle.

Some may argue that improving practitioner knowledge alone may not be enough. It relies on the individual to succeed despite a system that allows reporting to fall through the cracks, as opposed to a system succeeding despite imperfect individuals. Prior literature also notes that “administrative obstacles” were a major cause of nonreporting [9]. Previous work has noted that a Colorado telephone-reporting initiative more than doubled the number of reported gonorrhea cases [9]. This was suggested to be a result of a bimodal approach of correcting practitioner knowledge deficits as well as decreasing the previously mentioned administrative obstacles [9].

Having an immediate system-wide level intervention would certainly be effective in our project, but it is not feasible. This is why the first planned interventions are practitioner outreach and education.

This project has the potential to make a significant impact on the individual and systemic level. On the individual level, educating practitioners would lead to increased notification of affected patients and their partners, as well as confirmation of treatment. On the systemic level, fostering relationships between practitioners and the FLDOH could help reduce community rates of STDs/STIs through improved prevention and treatment. These aspects are both instrumental in reducing overall STD/STI transmission and improving overall public health.

Limitations

The generalizability of this project may be limited due to its focus on DOH-Manatee. These findings may not fully apply to other departments of health both within and outside of Florida, as well as regions with different organizational structures or practitioner networks.

Internal validity may have been affected by several factors. Although planning and development of the intervention was completed, it has not yet been distributed due to pending approval of the design and outreach plan by DOH-Manatee and FLDOH. Leadership turnover affecting internal operations, as well as a small survey response pool, may also limit representation of other perspectives. The survey was developed using input from DIS and was not externally validated, which may have introduced measurement bias by emphasizing internally identified issues while overlooking other relevant perspectives. Reliance on self-reported survey data introduced the potential for response bias. Furthermore, the transition from a planned focus group to a survey format reduced the depth of data to analyze. Limited access to DOH-Manatee data also restricted the ability to perform more detailed quantitative analysis and may have introduced measurement limitations.

To adjust for these limitations, a practical approach was taken by creating a low-cost, low-staff, digital outreach letter and educational email approach. This will reduce the burden on the DIS staff and would not cut into the DOH-Manatee budget. The materials created will require minimal intervention by the central office of the FLDOH for distribution approval, making it an efficient process.

## Conclusions

This project identified perceived barriers contributing to discrepancies between positive laboratory results and PDRF submissions, including potential gaps in practitioner awareness of reporting requirements and communication challenges with public health staff. These findings informed the development of a low-cost, sustainable digital outreach and practitioner education strategy to improve reporting practices.

Future evaluation should assess the effectiveness of the intervention using predefined outcome measures, including the ratio of STD/STI-positive laboratory results to PDRF submissions, with a goal of achieving greater than 50% reporting alignment, as well as improvements in the completeness and accuracy of submitted forms and timeliness of reporting. A repeat DIS survey may also be conducted to evaluate changes in perceived barriers following implementation. Additional monitoring will be needed to determine whether this approach improves reporting processes and can be adapted to other public health systems with similar reporting challenges.

## Appendices

**Table 1:** Survey Monkey questions sent to the disease intervention specialists OB/GYN = obstetrician-gynecologist; STDs/STIs = sexually transmitted diseases and infections

Question number	Question prompt	Response type
1	Overall, how would you rate your relationships with healthcare practitioners?	Likert Scale: 1=terrible, 2 =poor, 3= neutral, 4=good, 5=excellent
2	What strategies have been most effective in building practitioner relationships?	Open-ended
3	Have your relationships with maternal health practitioners (e.g., OB/GYNs, midwives) been different from your relationships with other practitioners (e.g., family medicine, emergency room, urgent care)?	Yes/No/Unsure
4	Please describe how these relationships differ.	Open-ended
5	If you have experienced challenges with practitioner relationships, what obstacles have you come across?	Open-ended
6	If you have had success with practitioner relationships, what actions contributed to that success?	Open-ended
7	Are you experiencing challenges with practitioners submitting the Practitioner Disease Report Forms (PDRFs)?	Yes/No/Unsure
8	Please describe the challenges.	Open-ended
9	Do you compare submitted PDRFs to positive lab results as part of your tracking process?	Open-ended
10	Have you noticed a gap between positive lab results and submitted PDRFs? If so, please explain.	Yes/No Open-ended for Yes
11	Where are most PDRFs being completed? Select all that apply: OB/GYN offices, Family medicine practices, community clinics, hospital emergency rooms, free-standing community clinics, urgent care centers, other (please specify)	Multiple Selections + Open-ended for “other”
12	How are clients with positive lab results but no confirmed treatment being tracked?	Open-ended
13	On average each month, how many clients with positive lab results do not have confirmed documentation of treatment?	Open-ended
14	Do you have difficulty getting the information you need from practitioners when following up on cases?	Yes/No
15	Please Describe the Difficulties	
16	How often do you need to send letters referencing Florida Statutes to request medical records from practitioners who are hesitant to share required information?	Open-ended
17	Please describe any common reasons practitioners give for not sharing the required records.	Open-ended
18	How is your Disease Interventionalist Specialist (DIS) team structured? Select all that apply: By case type (e.g., maternal vs. general STDs/STIs), All DIS handle all case types, Other (please specify)	Multiple Selections + Open-ended for “Other”

## References

[1] (2026). Bacterial sexually transmitted diseases (STDs). https://www.flhealthcharts.gov/ChartsDashboards/rdPage.aspx?rdReport=STD.Dataviewer&cid=9767.

[2] Hufstetler K, Llata E, Miele K, Quilter LA (2024). Clinical updates in sexually transmitted infections, 2024. J Womens Health (Larchmt).

[3] Garcia MR, Leslie SW, Wray AA (2026). Sexually transmitted infections. StatPearls.

[4] (2026). Practitioner disease report form. https://manatee.floridahealth.gov/wp-content/uploads/sites/42/2026/01/Disease-Report-Form.pdf.

[5] (2026). 381.0031 Epidemiological research; report of public health significance to department. https://www.leg.state.fl.us/Statutes/index.cfm?App_mode=Display_Statute&Search_String=&URL=0300-0399/0381/Sections/0381.0031.html.

[6] Ogrinc G, Davies L, Goodman D, Batalden P, Davidoff F, Stevens D (2016). SQUIRE 2.0 (Standards for QUality Improvement Reporting Excellence): revised publication guidelines from a detailed consensus process. BMJ Qual Saf.

[7] (2026). Health Insurance Portability and Accountability Act of 1996. https://www.congress.gov/104/plaws/publ191/PLAW-104publ191.pdf.

[8] Doyle TJ, Glynn MK, Groseclose SL (2002). Completeness of notifiable infectious disease reporting in the United States: an analytical literature review. Am J Epidemiol.

[9] Konowitz PM, Petrossian GA, Rose DN (1984). The underreporting of disease and physicians' knowledge of reporting requirements. Public Health Rep.

